# Mildly Higher Serum Prolactin Levels Are Directly Proportional to Cumulative Pregnancy Outcomes in *in-vitro* Fertilization/Intracytoplasmic Sperm Injection Cycles

**DOI:** 10.3389/fendo.2020.00584

**Published:** 2020-08-28

**Authors:** Duoduo Zhang, Xi Yuan, Jingran Zhen, Zhengyi Sun, Chengyan Deng, Qi Yu

**Affiliations:** ^1^Department of Obstetrics & Gynecology, Peking Union Medical College Hospital (PUMCH), Beijing, China; ^2^MOH Holdings (MOHH), Singapore, Singapore

**Keywords:** prolactin, GnRH agonist protocol, IVF, ICSI, cumulated clinical pregnancy rate, cumulated live birth rate

## Abstract

Hyperprolactinemia has long been considered detrimental to fertility due to irregularity of ovulation. Whether mild hyperprolactinemia should be corrected before initiating an *in-vitro* fertilization/intracytoplasmic sperm injection cycle (IVF/ICSI) has not been determined; this study aimed to examine how different levels of prolactin affect IVF outcomes. A total of 3,009 patients with basal prolactin level <50 ng/mL undergoing IVF/ICSI cycles for tubal or male factors were recruited in this study. Patients diagnosed with anovulation owing to polycystic ovarian syndrome or hyperandrogenism were ruled out. Pregnancy outcomes were compared between patients with basal prolactin levels higher or lower than the median level of prolactin (16.05 ng/mL). Multifactor analyses were carried out among four subgroups depending on different prolactin levels. Repeated-measures analysis of variance was used to explore the relationship between the ascending trend of prolactin levels over ovarian stimulation and the corresponding cumulative pregnancy outcomes. There were significantly higher numbers of oocytes (9 vs. 8, *P* = 0.013) and embryos (6 vs. 5, *P* = 0.015) in patients with basal prolactin higher than 16.05 ng/mL. Basal prolactin higher than 30 ng/mL was positively related to cumulative clinical pregnancy, and a level higher than 40 ng/mL was a good indicator for the cumulative live birth rate. Throughout ovarian stimulation, the prognosis of pregnancy improved with increasing prolactin levels. Patients with better cumulated pregnancy outcomes had significantly higher prolactin levels as well as a profoundly increasing trend during the stimulating process than those who did not conceive. For patients who underwent the gonadotropin-releasing hormone agonist long protocol IVF/ICSI treatment, a slightly higher prolactin level during the controlled ovarian hyperstimulation protocol was a positive indicator for cumulated pregnancy/live birth rates.

## Introduction

Prolactin (PRL) is known as a stimulator of the proliferation and differentiation of mammary cells for lactation. The primary regulator for PRL pituitary secretion is dopamine via hypothalamic inhibitory signals, and this constitutes the pharmacological basis for hyperprolactinemia treatment (1). In addition, PRL, as a stress hormone, is actively involved in metabolism, electrolyte transport, angiogenesis, and immunity (1).

Serum PRL is ordinarily under 25 ng/L; a level above the normal upper limit is diagnosed as hyperprolactinemia as long as the sample is obtained without excessive stress challenges before venipuncture. Hyperprolactinemia is a well-established cause of hypogonadotropic hypogonadism (2); PRL acts on kisspeptin-1 neurons expressing the PRL receptor (PRL-R) and is responsible for decreased kisspeptin-1 and GnRH secretion, leading to anovulation (3). Dopamine agonists are widely used for suppression of serum PRL and resumption of ovulation in infertile women with hyperprolactinemia seeking to conceive naturally. However, follicle genesis in women undergoing *in-vitro* fertilization/intracytoplasmic sperm injection (IVF/ICSI) treatment depends on exogenous gonadotropins, instead of endogenous ones, and luteal phase support is always ensured by sufficient progesterone (P) administration. Is it really necessary to suppress the slightly higher PRL? Or is there a proper PRL range to optimize IVF outcomes? We hypothesize an isolating mildly increasing PRL level if these women have no organic lesions such as prolactinoma would not negatively affect cumulated IVF pregnancy outcomes.

## Materials and Methods

This retrospective study included all women who underwent IVF/ICSI treatment for tubal or male infertility with the gonadotropin-releasing hormone agonist (GnRHa) long protocol at Peking Medical College Hospital (PUMCH) between 1st July 2014 and 31st March 2018. Patients diagnosed with anovulatory diseases like polycystic ovarian syndrome (PCOS) or hyperandrogenism were not included. The study was approved by the Ethics Committee of PUMCH (No. S-K601). Exclusion criteria were: Patients with serum P level ≥ 1.5 ng/mL during a controlled ovarian hyperstimulation protocol (COH), patients undergoing a freeze-all strategy, egg-donating cycles, basal PRL level ≥ 50 ng/mL, previous diagnosis of pituitary lesions, or abnormal thyroid functions. All patients included had to have used up all fresh or vitrified embryos generated from the stimulating cycle by the time of the study in order to analyze the cumulative pregnancy outcomes.

Sexual hormone levels were tested at three individual times for each patient. The first basal one was on the 2nd day of the menstrual cycle before pituitary downregulation by GnRHa, which we marked as T0. On the 2nd day of the next menstrual cycle patients started receiving recombinant human follicle-stimulating hormone (rFSH; Gonal-F, Merck-Serono) at an individualized dose adjusted based on patient ovarian response. Final oocyte maturation was triggered by intramuscular injection of 250 μg recombinant human chorionic gonadotropin (hCG; Ovitrelle, Merck-Serono) and the 2nd hormone sample taken on that morning was defined as T1. The third hormone samples were collected on the morning after administering hCG, and that time was defined as T2. After that, oocytes were retrieved by ultrasound-guided transvaginal aspiration at around 36 h after hCG trigger. Intramuscular injection of 40 mg P was administered daily for luteal phase support. Embryo development was evaluated daily until the fresh transfer of cleavage stage embryos (Day 3). Embryos were evaluated following a standardized scoring system (4). After fresh embryo transfer, the remaining embryos were cultured to blastocysts (Day 5 or 6) before vitrification. Frozen-thawed embryo transfer may be applied to either artificial or natural cycles.

Serum FSH, luteinizing hormone (LH), PRL, estrogen (E_2_), and P levels were measured by the automated Elecsys Immunoanalyzer (Beckmann, USA). The inter-assay coefficients of variation were <5 and <10% for E_2_ and P and <8% for FSH, LH, and PRL, respectively.

Clinical pregnancy was defined as intrauterine pregnancy with at least one fetus with a positive heartbeat at 6 weeks of gestation or later. Live birth was defined as the delivery of a live-born child at >28 weeks of gestation. The clinical pregnancy rate (CPR) and live birth rate (LBR) referred to the cumulated outcome after transferring all embryos from the studied stimulating cycle. Secondary outcomes included the number of oocytes retrieved, mature oocytes, two-pronuclear zygotes, and embryos.

The data analysis was carried out using SPSS 24.0 statistical analysis software (IBM Inc., USA). The normality of distribution of continuous variables was assessed using the Kolmogorov-Smirnov test (cutoff at *P* = 0.01). Descriptive statistics for continuous variables are reported as the mean ± standard deviation (SD). Categorical variables were described using frequency distributions and are presented as frequency and percentage (%). The *t*-test for independent samples or the Mann-Whitney *U*-test were used as appropriate to compare continuous variables by group. The chi-squared test was used to compare categorical variables by group. Repeated-measures analysis of variance was used for measuring repeated longitudinal data. A logistic regression model of the two groups (PRL ≤ 16.05 vs. PRL > 16.05 ng/mL) was developed to additionally adjust for age, body mass index (BMI), basal FSH, basal E_2_, and duration of infertility. Odds ratios were estimated with 95% confidence intervals. All tests were two-sided and considered significant at *P* < 0.05.

## Results

**Figure 1:**
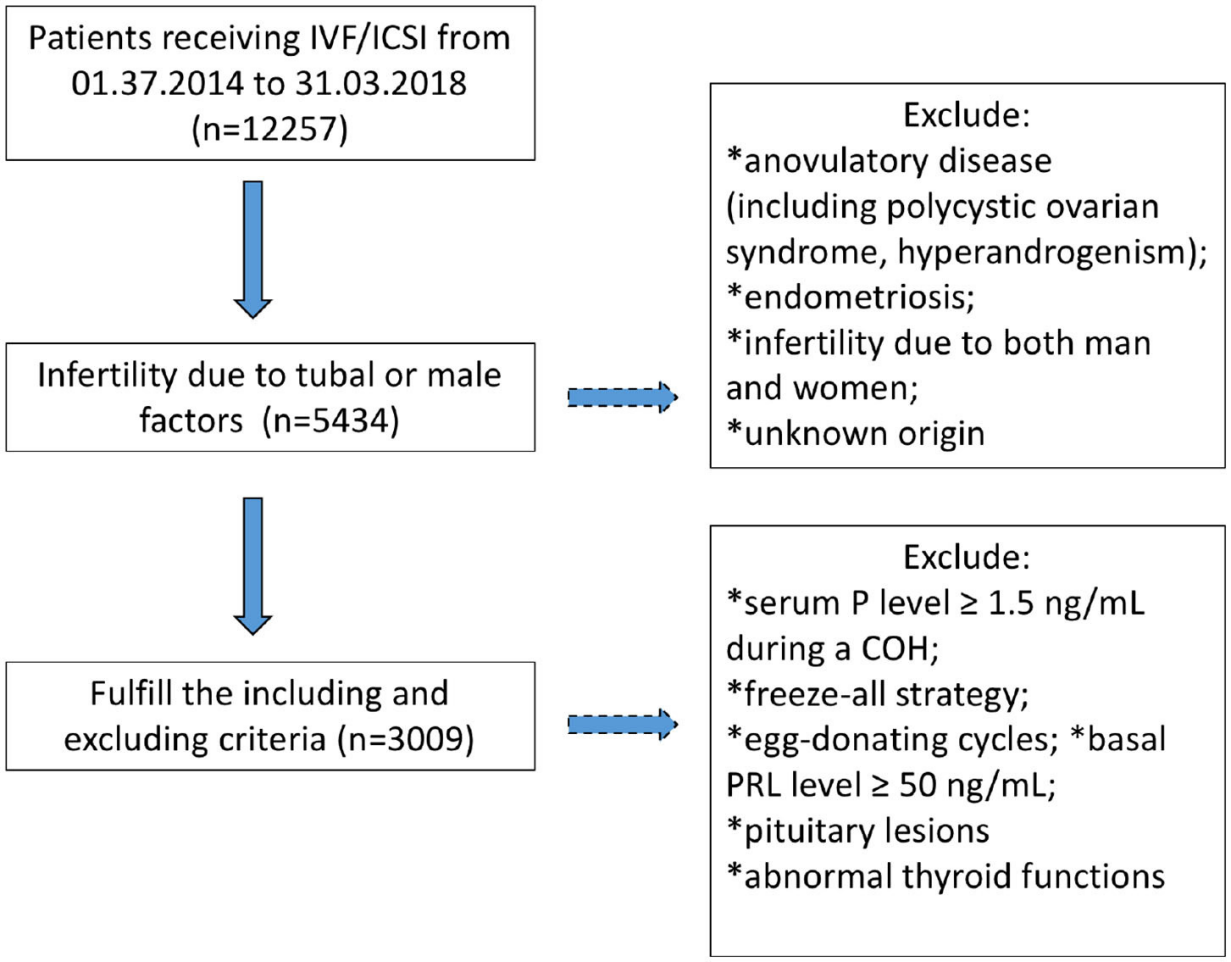
Flowchart regarding patients' inclusion and exclusion. COH, controlled ovarian hyperstimulation; P, progesterone; PRL, prolactin.

A total of 3,009 patients fulfilling the criteria were recruited in the study, of whom, 2098 underwent IVF cycles and 911 received ICSI (Figure 1). Their demographic characters were shown in Table 1. To explore the relationship between basal PRL (T0) and pregnancy outcomes, we divided patients into two groups by median PRL level (≤16.05 vs. >16.05 ng/mL). The two groups were compared in terms of baseline characteristics and pregnancy outcomes (Table 1). Patients with basal PRL >16.05 ng/m had slightly but significantly more oocytes retrieved, MII oocytes, fertilization, and embryos (*P* < 0.05). No statistically significant differences in cumulated CPR and LBR were detected between the two groups.

**Table 1:** Comparisons of baseline characteristics and pregnancy outcomes between patients with PRL ≤ 16.05 or > 16.05 ng/mL.

	**PRL ≤ 16.05 ng/mL (1506)**		**PRL > 16.05 ng/mL (1503)**		***P***
Age (years)	35.215	4.391	34.613	4.257	0.079
BMI (kg/m^2^)	22.364	3.186	21.835	3.063	0.102
Duration of infertility (years)	4	2, 6	4	3, 6	0.978
**Basal sexual hormone**					
FSH (IU/L)	7.130	5.770, 9.000	7.190	5.930, 9.010	0.343
LH (IU/L)	3.655	2.520, 5.093	3.840	2.730, 5.230	0.193
E_2_ (pg/mL)	45.910	34.985, 58.730	46.240	35.320, 60.260	0.344
rFSH starting dose (ampoule)	4	3, 4	4	3, 4	0.608
Oocytes retrieved	8	5, 11	9	5, 12	0.013
MII oocytes	6	3, 10	7	4, 10	0.008
Zygotes	5	3, 9	6	3, 10	0.012
Embryos	5	3, 9	6	3, 10	0.015
Cumulative CPR	50.1%	755/1506	53.9%	810/1503	0.097
Cumulative LBR	44.5%	670/1506	47.3%	711/1503	0.065

*Continuous variables following the normal distribution are presented as the mean (SD); non-normal distribution parameters are presented as the median (quartile); categorical variables are presented as percentages (with their frequencies)*.

We further divided all patients into five groups according to different basal PRL levels: Group I with PRL 0–9.9 ng/ml, Group II, 10–19.9 ng/ml; Group III, 20–29.9 ng/ml; Group IV, 30–39.9 ng/ml; and Group V, 40–49.9 ng/ml (Table 2). Nearly half of the patients were distributed in Group II. Therefore, we applied Group II as a dummy variable. Other factors including age, basal FSH, rFSH starting dose, total consumption of rFSH, basal E_2_, and BMI were entered into the multifactor analysis. It turned out the last two factors (basal E_2_ and BMI) were not statistically significant. The results revealed that a higher basal PRL was related to a better rate of cumulated clinical pregnancy and live birth.

**Table 2:** Multifactor analysis of the relationship between basal PRL and pregnancy outcomes.

**Outcomes**	**Group**	**Basal PRL (ng/mL)**	**%**	**Frequency (3,009 in total)**	***P***	**OR**	**95% CI**
Cumulative clinical pregnancy	I	0–9.9	13.5	407	0.047	0.858	0.683, 0.879
	II	10–19.9	56.1	1,689	–	1.000	–
	III	20–29.9	21.8	655	0.569	0.997	0.823, 1.207
	IV	30–39.9	6.4	192	0.046	1.281	1.030, 1.764
	V	40–49.9	2.2	66	0.039	1.639	1.247, 2.837
Cumulative live birth	I	0–9.9	13.5	407	0.047	0.871	0.691, 0.997
	II	10–19.9	56.1	1,689	–	1.000	–
	III	20–29.9	21.8	655	0.354	1.030	0.850, 1.247
	IV	30–39.9	6.4	192	0.341	1.139	0.830, 1.562
	V	40–49.9	2.2	66	0.008	1.916	1.115, 3.290

*Categorical variables are presented as percentages (with their frequencies), OR, and 95% CI. The clinical pregnancy rate and live birth rate are additionally adjusted for age, bFSH, rFSH starting dose, and total consumption of rFSH*.

In order to analyze the fluctuation of PRL levels through COH in the GnRHa long protocol cycle and to examine whether the change in PRL level is related to IVF pregnancy outcomes, we compared the PRL levels between patients with positive and negative pregnancy results on T0 (basal status), T1 (end of follicular stage), and T2 (early initiation of luteal phase) (Tables 3, 4). There were 1,585 cases with positive cumulated clinical pregnancy and 1,381 cases with cumulated live birth. The PRL levels of patients with positive pregnancy outcomes were significantly higher at all measurement points than those of patients with negative results. Moreover, a sharper spike was observed in groups with positive clinical pregnancy or live birth.

**Table 3:** Comparison of serum PRL levels between different pregnancy outcomes at different time points by repeated-measures analysis of variance.

**Pregnancy outcomes**	**T0 (ng/mL)**	**T1 (ng/mL)**	**T2 (ng/mL)**
Cumulative clinical pregnancy	16.21 (12.34, 22.05)	32.7 (23.92, 43.86)	33.16 (24.38, 46.69)
No clinical pregnancy	15.85 (11.62, 21.15)	27.46 (19.29, 38.76)	30.12 (20.31, 42.06)
*P*-Value	0.011	<0.001	<0.001
Cumulative live birth	16.25 (12.34, 22.08)	33.06 (24.31, 43.82)	33.45 (24.61, 46.475)
No live birth	15.85 (11.74, 21.23)	27.84 (19.56, 39.47)	30.31 (20.67, 42.42)
*P*-Value	0.020	<0.001	<0.001

*Non-normal distribution parameters are presented as median (quartile)*.

**Table 4:** Absolute difference of serum PRL between different time points.

**Time point**	**Group**	**Positive**	**Negative**	***P***
**for**		**outcomes**	**outcomes**	
**comparison**		**(ng/mL)**	**(ng/mL)**	
Δ_1_PRL (T1–T0)	Cumulative clinical pregnancy	13.297 (15.096)	17.416 (15.959)	<0.001
	Cumulative live birth	13.719 (15.330)	17.528 (15.852)	<0.001
Δ_2_PRL (T2–T1)	Cumulative clinical pregnancy	2.534 (9.962)	1.720 (10.565)	0.030
	Cumulative live birth	2.572 (9.990)	1.555 (10.611)	0.007

*Continuous variables following the normal distribution are presented as the mean (SD)*.

## Discussion

In this retrospective study, we analyzed the relationship between basal PRL levels, as well as their increasing tendency, and pregnancy outcomes of IVF/ICSI treatments for tubal/male factor infertility. Hyperprolactinemia has long been considered detrimental to fertility due to its effect on blocking LH secretion, leading to anovulation, or luteolysis (2). However, in IVF, oocyte maturation is induced by hCG trigger, and sufficient luteal phase support is guaranteed by progesterone supplements. Therefore, IVF procedures provide an ideal opportunity to observe the potential effect of PRL on reproduction in comparison to suppression of gonadotropins. This study was designed to answer two main questions: [1] Are cumulative pregnancy outcomes better in women with higher basal PRL levels when it is under 50 ng/mL?; [2]. In cycles with better pregnancy outcomes, will there be greater increase of PRL throughout ovarian stimulation (basal state, hCG day, and the day after hCG triggering)?

Around 85% of PRL molecules in circulation are 23 kDa monomers, which is the major bioactive form of PRL. Approximately a quarter of patients with hyperprolactinemia are shown to have macroprolactinemia. Women with macroprolactinemia may have no symptoms despite their elevated serum PRL levels due to inactive macroprolactin (5). That is to say, some asymptomatic hyperprolactinemia may be caused by macroprolactinemia; thus, such patients may not need dopamine agonist administration before IVF treatment. However, macroprolactin was not measured in our study. Future research should study macroprolactin and the proportion of active PRL levels.

Kamel et al. found that women who conceived had a remarkable increase of PRL compared to women who did not conceive, supporting the variation we found between T2 vs. T1 and T1 vs. T0. Additionally, higher PRL levels were associated with higher embryo quality (6). In our study, PRL levels were noted to increase throughout the COH. Unlike the high PRL levels because of ovarian stimulation, pre-existing hyperprolactinemia before IVF/ICSI treatment puzzles physicians the most. Doldi et al. prescribed cabergoline as pretreatment to women with hyperprolactinemia until egg-retrieval. Thus, the PRL levels were significantly lower than those of the control group who did not receive cabergoline. However, there was no improvement of CPR in patients treated with cabergoline adding the effect of rFSH consumption (38.1 ± 18.2 vs. 43.9 ± 28.5 ampoule; *P* < 0.05), lower MII oocyte rate (87.9 vs. 80.4%; *P* < 0.05), and fertilization rate (70.8 vs. 60.8%; *P* < 0.03) (7).

It is known that better IVF/ICSI outcomes are observed in patients with higher PRL levels in either the basal state or during COH. According to the present findings, the group with basal PRL level > 16.05 ng/mL experienced a surge in the numbers of oocytes, MII oocytes, zygotes, and embryos. Previous research by Mendoza et al. discovered that higher basal PRL levels are related to larger numbers of mature oocytes and good quality embryos (8), suggesting that PRL plays a role in oocyte maturation as well as embryonic development. Oogenesis is a complicated process involving oocytes and the granular cell cumulus actively exchanging signals within the circulating body fluid. Nakamura et al. reported that PRL receptor-knocked-out mice can only produce eggs with intact germinal vesicles (9). In contrast, higher mature rates were found when exogenous PRL was added to pre-antral follicle cultures of the IVF system (10). It could be a possible hypothesis that a certain PRL level guarantees the accomplishment of meiosis. Moreover, in the PRL receptor in deprived mice, there was a sharp decrease of the fertilization rate; most of the zygotes underwent retardation, and only 19% developed to blastocysts (11). PRL participates in embryo implantation via BRCA1, a protein expressed on the surface of the trophoblast cells. As the PRL concentration gradually increased in the pre-antral follicle culture (0, 10, 20 mIU/mL), BRCA1 expression also increased (12). Although there was no statistical significance, there was an increasing trend of the implantation rate from 47.0% in the control population to 56.1% when cultured with 20 mIU/mL PRL (12). Since PRL improved oogenesis and embryonic development, some researchers have tried to improve the IVF outcomes by prescribing bromocriptine to patients with a history of recurrent implantation failure until the initiation day of rFSH. Therefore, PRL rebounded to a higher level, and the CPR did improve compared to that in the controls (10.1 vs. 27.2% *P* < 0.05) owing to the significantly increased PRL (13).

In addition to its effect on oogenesis and embryogenesis, PRL also boosts other physiological reproductive activities. When either PRL genes or PRL-receptor genes were knocked out, a profound decrease in progesterone levels was noticed in the luteal phase of mice (11); moreover, the corpus luteum underwent early degradation 2 days after mouse intercourse (14). We revealed that a dramatic surge of PRL after luteinization was associated with better cumulated IVF/ICSI outcomes. This is consistent with the promoting effect of PRL on luteal function (15). PRL stimulates the long chain receptor in the luteinized cells to activate the Jak2/STAT5 pathway and suppress 20-α-hydroxysteroid dehydrogenase, subsequently spurring progesterone production. Meanwhile, multiple vascular endothelial growth factors are secreted into the ovaries to accelerate the vascularization of the corpus luteum when the PRL short chain receptor is stimulated. A human study by Daly et al. raised the concern that mid-luteal PRL levels were the lowest (15.0 ± 11.7 ng/mL) in women with early pregnancy loss compared to those who were infertile or expected to conceive normally (16). Furthermore, PRL acts on the adaptive immune system. PRL receptors are widely expressed on the surface of CD4+ T cells and B cells. Once stimulated, inflammatory factors such as interleukin-2 and interferon-gamma would be suppressed (17). This process might allow an immune privilege status between the maternal-fetal interface leading to a smoother pregnancy.

The major limitation of our study is that no causal relationship between PRL and IVF pregnancy outcomes could be inferred due to the study's retrospective nature. In multifactor regression, we found that the pregnancy outcomes became better as PRL increased. Nevertheless, the power of the test may be compromised due to the significantly different number of patients in each subgroup and the number of patients with hyperprolactinemia decrease with increasing basal PRL levels. Reasonably, the beneficial effect of PRL cannot continue permanently and constantly rising, and there should be an inflection point of PRL level beyond which, the advantageous effect on IVF/ICSI pregnancy outcomes would become harmful. However, in clinical practice, physicians are prone to prescribe dopamine agonists to patients with high PRL level > 50 ng/mL before entering a cycle; thus, we could not recruit such patients. Consequently, the inflection point could not be illustrated by our recruited sample. In this study, we targeted mainly at tubal or male factor infertility. Particularly we avoided including anovulation or endometriosis because these diseases possibly interfere with ovarian reserve or HPO axis and, in turn, affect the PRL status. For example, PCOS or hyperandrogenemia was both sorted to anovulatory disorders in our center and excluded. However, if a patient had not meet the full diagnosis criteria of PCOS, but merely demonstrated either a polycystic ovarian morphology or very mild hyperandrogenemia which appear not to interfere with regular ovulation, she could still be included as long as she was sorted as tubal or male factor infertility. This could lead to a potential bias since PCOS or hyperandrogenemia will slightly increase the PRL level. Another drawback of this study is we merely employed basal FSH as the major ovarian reserve indicator. As we know, ovarian reserve markers are closely related to the number of oocytes retrieved as well as the CCPR and CLBR. However, our center has not initiated universal AMH test until 2019, and the data of AFC are not uniformly documented. Luckily we will have had enough AMH data to analyze in foreseeable future.

In conclusion, for patients receiving IVF/ICSI treatment with a basal PRL level within the range of 0–50 ng/mL, higher PRL levels were associated with higher numbers of oocytes, mature oocytes, zygotes, and embryos. Both the cumulative CRP and LBR increased with increasing PRL levels. There was a remarkable surge of PRL level from the basal status to the next day after hCG injection. The beneficial effect of PRL on pregnancy outcomes may be attributed to the facilitation of oogenesis and embryonic development, as well as the improvement of luteal function. Hence, in clinical settings, when physicians encounter a patient with asymptomatic hyperprolactinemia planning IVF/ICSI treatment, the serum PRL level may be not suppressed to an extremely low level if organic lesions are excluded.

## Data Availability Statement

The datasets generated for this study are available on request to the corresponding author.

## Ethics Statement

The studies involving human participants were reviewed and approved by the Ethics Committee of PUMCH (No. S-K601). The patients/participants provided their written informed consent to participate in this study.

## Author Contributions

DZ analyzed the data and drafted the manuscript. XY designed the study and revised the manuscript. DZ and XY did a major and equal contribution to this work. ZS was responsible for the data acquisition. CD analyzed and interpreted the data. QY edited the manuscript. JZ provided final approval for the submitted version and is responsible for the whole work. All authors have critically reviewed and approved the final submitted version.

## Conflict of Interest

The authors declare that the research was conducted in the absence of any commercial or financial relationships that could be construed as a potential conflict of interest.

## Abbreviations

BMI: body mass index
CPR: clinical pregnancy rate
COH: controlled ovarian hyperstimulation
FSH: follicle stimulating hormone
GnRHa: gonadotropin releasing hormone agonist
hCG: human chorionic gonadotropin
LBR: live birth rate
ICSI: intracytoplasmic sperm injection
IVF: *in vitro* fertilization
PRL: prolactinl
rFSH: recombined human FSH.
